# GLP-1 Relaxes Rat Coronary Arteries by Enhancing ATP-Sensitive Potassium Channel Currents

**DOI:** 10.1155/2019/1968785

**Published:** 2019-10-23

**Authors:** Qian-Feng Xiong, Shao-Hua Fan, Xue-Wen Li, Yu Niu, Jing Wang, Xin Zhang, Yi-Fan Chen, Ya-Wei Shi, Li-Hui Zhang

**Affiliations:** ^1^Department of Cardiology, Fengcheng People's Hospital, Fengcheng 331100, Jiangxi, China; ^2^Key Laboratory of Chemical Biology and Molecular Engineering of Ministry of Education, Institute of Biotechnology, Shanxi University, Taiyuan 030006, Shanxi, China; ^3^Department of Cardiology, Shanxi Dayi Hospital Affiliated to Shanxi Medical University, Taiyuan 030024, Shanxi, China; ^4^Department of Geriatrics, Shanxi Dayi Hospital Affiliated to Shanxi Medical University, Taiyuan 030024, Shanxi, China

## Abstract

GLP-1 is a new type of antidiabetic agent that possesses many beneficial effects. Although its cardiovascular actions have been widely examined, little is known about GLP-1's effects on the rat coronary artery (RCA) or about the mechanisms underpinning these effects. Here, we report that GLP-1 inhibits depolarization- or thromboxane receptor agonist (U46619)-induced RCA contraction in a dosage-dependent manner. Vasorelaxation was attenuated by denuding the endothelium, L-NAME (nitric oxide synthase inhibitor), and glyburide (K_ATP_ channel blocker) but was not affected by indomethacin (cyclooxygenase inhibitor), iberiotoxin [Ca^2+^-activated K^+^ channel (K_Ca_) blocker], or 4-aminopyridine (K_V_ channel blocker). Furthermore, GLP-1 increased outward K^+^ currents by enhancing the K_ATP_ channel in rat coronary arterial smooth muscle cells (RCASMCs). These results show that GLP-1 is an endothelial-dependent vasospasmolytic agent in the RCA and imply that the relaxant effect is regulated by enhancing K_ATP_ rather than K_V_ or K_Ca_ currents in RCASMCs.

## 1. Introduction

Glucagon-like peptide-1 (GLP-1) is an incretin synthesized and secreted by intestinal L cells and a peptide that binds to its GLP-1 receptor (GLP-1R) to regulate glucose homeostasis by stimulating insulin secretion in a glucose-dependent manner. GLP-1 analogues, such as exenatide, liraglutide, and dulaglutide, are currently in use for treating type 2 diabetes mellitus. As GLP-1R is widely expressed in many organs, including the pancreatic islets, heart, vessels, kidneys, and brain [1], the pleiotropic effects of GLP-1 have become a very promising research topic. Accumulating studies suggest that GLP-1 plays a cardioprotective role by regulating glucose uptake and left ventricular function [2]. The LEADER (Liraglutide Effect and Action in Diabetes: Evaluation of Cardiovascular Outcome Results) trial demonstrated that, beyond its original role as an antidiabetic agent, the GLP-1 receptor agonist liraglutide exerts a blood pressure-lowering effect in addition to reducing the cardiovascular disease risk [3]. In our previous study, the antihypertensive effect of GLP-1 was observed in a rat model with an implanted angiotensin II-infusion osmotic pump [4]. Furthermore, experimental evidence on the *ex vivo* vascular actions has revealed that GLP-1 (7–36), an active isoform of GLP-1, exerts a vasorelaxant action on different types of blood vessels, such as the rat thoracic aorta [5, 6], porcine ileal artery [7], rat pulmonary artery [8], and femoral artery [9].

Animal studies have demonstrated that GLP-1 (7–36) increases coronary flow in constant pressure-perfused isolated hearts in wild-type and Glp-1r^−/−^ mice [1]. Moreover, augmented coronary flow has been observed a short time after GLP-1 intervention in an isolated rat heart, but there was no variation in the left ventricular diastolic pressure or heart rate [10]. Therefore, we hypothesized that GLP-1 exerts a direct vasodilative effect on the coronary artery to increase coronary flow and further improve myocardial function.

ATP-sensitive potassium (K_ATP_) channels, one of the most important potassium channels, have been reported in a variety of tissues and organs, including the pancreas, heart, and smooth muscle [11]. In the vascular smooth muscle, the K_ATP_ channel is involved in adjusting vasoconstriction and relaxation by regulating intracellular Ca^2+^ concentrations [12]. Until recently, no study has explored the effect of GLP-1 on K_ATP_ channels in rat coronary arterial smooth muscle cells (RCASMCs) using the patch clamp technique.

In the present study, we investigated the potential vasorelaxant effects of GLP-1 on the rat coronary artery and examined the mechanisms underlying these effects by investigating the impact of GLP-1 on the K_ATP_ currents in RCASMCs.

## 2. Materials and Methods

### 2.1. Reagents

GLP-1 was synthesized by Sangon Biotech, and NG-nitro-L-arginine methylester ester (L-NAME), indomethacin, 4-aminopyridine (4-AP), glyburide (Glyb), iberiotoxin (Iber), 9,11-dideoxy-9*α*,11*α*-methanoepoxy prostaglandin F2*α* (U46619), and HEPES were purchased from Sigma.

### 2.2. Animals

All animals (male Wistar rats: weight, 280–330 g; age, 6 months) were purchased from the Animal Facility Center of Shanxi Medical University, China. All experiments in this study were conformed to the Animal Experimentation Ethics Committee of Shanxi Medical University. Rat coronary arteries with an inner diameter of 240–330 *μ*m were used for the myogenic study and cell isolation.

### 2.3. Artery Ring Preparations

All rats were anesthetized with an intraperitoneal injection of sodium pentobarbital (40 mg/kg). After euthanizing the rats by exsanguination, the heart was removed and transferred immediately to a chilled (4°C) physiological salt solution (in mmol/l: 5 HEPES, 11 D-glucose, 144 NaCl, 2.5 CaCl_2,_ 5.8 KCl, and 1.2 MgCl_2,_ pH 7.4) [13]. Rat coronary arteries were isolated bluntly and subsequently used to prepare arterial rings of 2 mm in length under the view of a dissecting microscope (Olympus SZ51, Tokyo, Japan). Two 40 *μ*m diameter tungsten wires were inserted into the rings and fixed to the Multi-Myograph System-610M Microvascular Tension Recorder (Danish Myo Technology A/S, Aarhus, Denmark). The ring bath, containing 5.0 ml of physiological salt solution, was bubbled with 100% O_2_ and maintained at 37°C. The rings were adjusted to a state equal to 80 mmHg according to standardized procedures. After 1 h of equilibration, the rings were contracted repeatedly with 60 mM KCl and relaxed with acetylcholine. We picked out the arterial rings that met the following conditions: (1) the contraction activated by 60 mM KCl was more than 2 mN (2) and the contraction was constant or repeatable [14]. After each contraction, the next experiment can be performed until the arterial ring tension is restored. We allowed the time interval between two experiments to be around 1 hour.

### 2.4. Effects of Pretreatment with GLP-1 on KCl- or U46619-Induced Contraction

After the rings stabilized, U46619 (0.03, 0.1 0.3, 1, and 3 *μ*M) or KCl (20, 28, 39, 55, 77, and 108 mM) were added to the bath to cause coronary vascular ring contractions in a concentration-dependent manner. When the rings were in a stable state, the next concentration of the two chemicals mentioned above was added. After qualified contraction curves can repeat fluently, GLP-1 was added to the bath and preincubated for 15 min. In the absence of GLP-1, the contraction value caused by the maximum concentration of KCl or U46619 was regarded as 100%, and the percentage of contraction of KCl or U46619 was calculated and then used to build the curves.

### 2.5. Effects of GLP-1 on Precontractions

GLP-1 was added to the bath at cumulatively increasing concentrations (0.001, 0.01, 0.1, 1, 10, and 100 *μ*M) when KCl- (60 mM) or U46619- (1 *μ*M) induced vascular ring contraction experiments can be repeated successfully. When the relaxation effect of a certain concentration of GLP-1 was stabilized, the next GLP-1 concentration was added to the bath. The maximal contraction caused by KCl (60 mM) or U46619 (1 *μ*M) was regarded as 100%, and then we calculated the percentage of relaxation by different concentrations of GLP-1.

### 2.6. Effects of Denudation and Inhibitors on GLP-1-Induced Relaxation of RCAs

The effects of different inhibitors on GLP-1-induced relaxation were studied to investigate the possible mechanisms underlying this phenomenon. The following inhibitors were used in the experiment: 10 *μ*M indomethacin, a cyclooxygenase inhibitor; 0.1 mM L-NAME, a nitric oxide synthase inhibitor; 1.0 mM 4-AP, a K_V_ channel blocker; 0.1 *μ*M Iber, a Ca^2+^-activated K^+^ channel (K_Ca_) blocker; and 1 *μ*M Glyb, a K_ATP_ channel blocker. When the contraction induced by 1 *μ*M U46619 was repeatable, one of these inhibitors was added to the bath. GLP-1 was added to the bath as vascular tone in the presence of a stabilized inhibitor. Relaxation was expressed as a percentage of the precontraction induced by 1 *μ*M U46619. The effect of GLP-1 in denuded rings was observed to explore the involvement of the endothelium in GLP-1-caused vasorelaxation. The method of removing the endothelium was as follows: a fine tube was inserted into the RCA ring and bubbles were injected gently several times [15]. After the vasoactivity was detected according to the aforementioned method, 1 *μ*M acetylcholine was added to the bath. We considered the endothelium was denuded when the arterial rings relaxation induced by acetylcholine was <40% [16].

### 2.7. Cell Isolation

After separating the coronary arteries, coronary vascular smooth muscle cells were obtained by two-step enzymatic hydrolysis. First, the RCAs were dissected into HEPES solution (1 mg/ml albumin, 0.1 mM CaCl_2,_ 1 mg/ml dithioerythritol, and 0.5 mg/ml papain) and incubated for 30 min at 37°C. Second, the RCAs were transferred to Ca^2+^-free HEPES solution and incubated for 15 min at 37°C [17]. Finally, a Pasteur pipette was used to gently triturate a single cell from the vascular ring. The enzymatic cell suspension was centrifuged (1,000 rpm; 10 min), the supernatant was discarded, and calcium-free HEPES solution was slowly added. The cells were centrifuged three times to remove debris and residual enzymes [18]. Isolated RCASMCs were used immediately for electrical recording.

### 2.8. Electrophysiological Measurements

Transfected RCASMCs on glass cover slips were placed in an experimental bath with HEPES solution attached to the stage of an inverted microscope (Nikon ECLIPSE TE2000-S, Tokyo, Japan). We used a computer-controlled clamp system driven by Clampex10.3 for the whole-cell voltage clamp and Clampfit10.3 for experiment data analysis. During the patch clamp experiment, series resistance, pipette offset, and cell capacitance were compensated for electronically [17]. The cell capacity was 7–15 pF, and the resistance of the electrodes was 3–5 MΩ when filled with pipette solution. Current traces were percolated at 1 kHz with Bessel ﬁlter in the clamp ampliﬁer, digitized at 5 kHz, and then stored on storage medium for data analysis. The bath solution used to record K_ATP_ currents contained (mM) 138 NaCl, 5.6 KCl, 1.2 MgCl_2_, 2.6 CaCl_2_, and 2 HEPES, pH 7.4. The patch pipette solution contained (mM) 76 K_2_SO_4_, 10 KCl, 1 MgCl_2_, 10 NaCl, and 10 HEPES, pH 7.4 [19]. The pipette solution eliminated any K_V_ or K_Ca_ currents. The cells were held at a potential of −140 mV. We allowed cells to perform a step depolarization of 500 ms to −30 mV in increments of 10 mV per second.

### 2.9. Data Analysis

All group values are expressed as mean ± standard deviation. GraphPad Prism 7 software was used to detect differences. Differences between two groups were analyzed by Student's *t-*test, and one-way analysis of variance was used when more than two groups were compared. A *P* value <0.05 was considered significant. The values of IC_50_ (half-maximal inhibitory concentration) and EC_50_ (concentration for 50% of maximal effect) were determined by linear regression.

## 3. Results

### 3.1. Effects of Pretreatment with GLP-1 on KCl or U46619 Concentration-Contraction Curves

**Figure 1:**
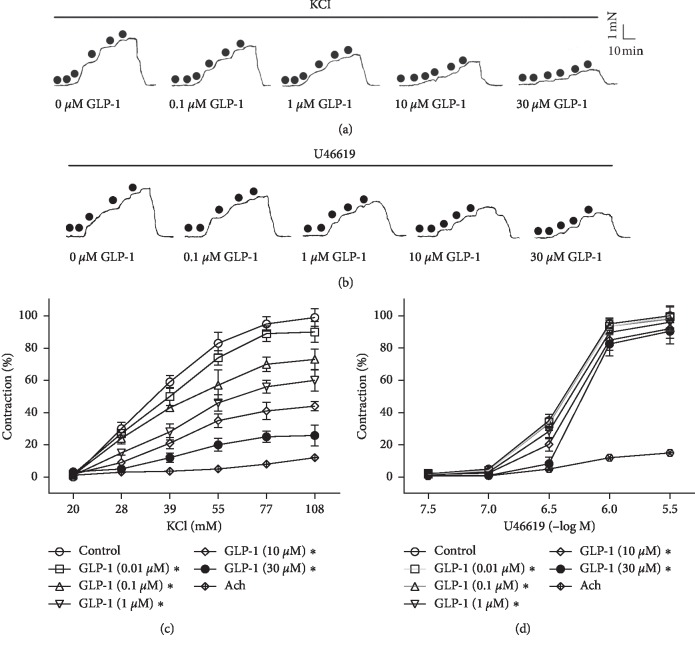
Effect of glucagon-like peptide-1 (GLP-1) on the KCl or U46619 concentration-contraction curve. The myogenic response to the increase in KCl or U46619 was recorded in the absence or presence of GLP-1 (0.01, 0.1, 1, 10, and 30 *μ*M) in rat coronary artery (RCA) rings (240–330 *μ*M in inner diameter). (a, b) Typical original tension tracings. (c, d) Relaxation is expressed as a percentage of the maximal contraction induced by 108 mM KCl or 3 *μ*M U46619, respectively. Data are mean ± standard deviation; *n* = 6; ^*∗*^*P* < 0.05 vs. control.

KCl (20–108 mM) or U46619 (3 × 10^−8^–3 × 10^−6^ M) concentration dependently contracted the RCA. The maximal contractions were 4.73 ± 0.44 mN for KCl and 4.61 ± 0.35 mN for U46619. The EC50 values were 37.13 mM and 0.35 *μ*M, respectively (Figures 1(a) and 1(b)). Both of the above concentrations are much higher than the physiological level of GLP-1. Healthy subjects with normal glucose tolerance had a fasting plasma GLP-1 level of 4.9 ± 0.4 pmol/liter [20]. After preincubation with GLP-1, the two concentration-contraction curves moved to the lower right, and the IC50 values were 8.45 *μ*M for KCl and 13.24 *μ*M for U46619. GLP-1 decreased the maximal contraction by 73.13% for KCl and by 9.55% for U4619 (Figures 1(c) and 1(d)).

### 3.2. Effects of GLP-1 on Precontractions Induced by KCl or U46619

**Figure 2:**
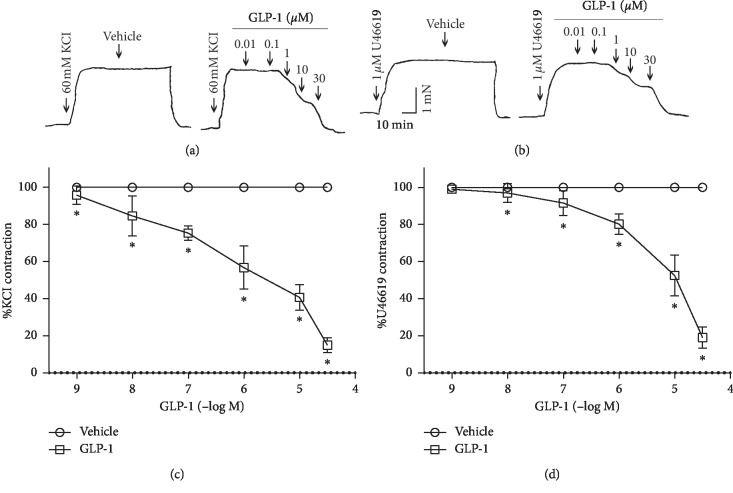
Glucagon-like peptide-1- (GLP-1-) induced relaxation in rat coronary artery (RCA) rings precontracted with KCl or U46619. (a, b) Typical original tension recording. (c, d) Relaxation is expressed as a percentage of the precontraction induced by 60 mM KCl or 1 *μ*M U46619, respectively. Data are mean ± standard deviation; *n* = 6; ^*∗*^*P* < 0.05 vs. control.

To investigate the vasodilatory effect of GLP-1 on the RCA, GLP-1 was gradually added to the bath to reach the target concentration (0.01, 0.1, 1, 10, or 30 *μ*M) when the contraction mediated by KCl (60 mM) or U46619 (1 *μ*M) was stabilized. GLP-1 inhibited depolarization (Figures 2(a) and 2(c)) and U46619 induced (Figures 2(b) and 2(d)) contractions in a dose-dependent manner.

### 3.3. Effects of Denudation and Inhibitors on GLP-1-Induced RCA Relaxation

**Figure 3:**
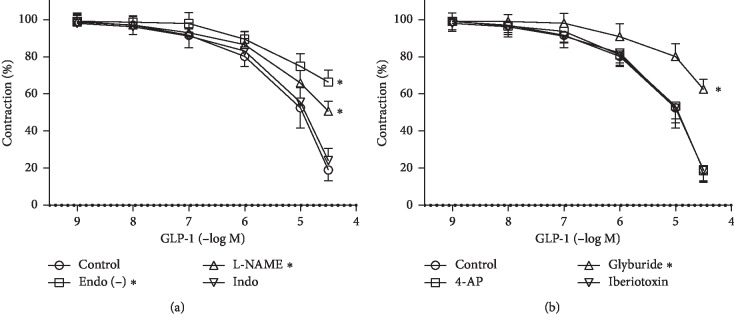
Effects of inhibitors on glucagon-like peptide-1- (GLP-1-) induced relaxation in the rat coronary artery (RCA). The rings were precontracted with 1 *μ*M U46619. After the precontraction was sustained and steady in endothelium-preserved rings, L-NAME (0.1 mM), indomethacin (0.01 mM), 4-AP (1 mM), glyburide (1 *μ*M), or iberiotoxin (0.1 *μ*M) was added to the bath. When the contraction was steady again in the presence of one of the inhibitors, the GLP-1 concentration-relaxation curve was reconstructed. The relaxation of endothelium-denuded rings by GLP-1 was compared with that of endothelium-preserved rings isolated from the same rat. Relaxation is expressed as a percentage of precontraction. Data are mean ± standard deviation; *n* = 6; ^*∗*^*P* < 0.05 vs. control.

Cyclooxygenase (COX) is an enzyme responsible for the formation of prostanoids which have relaxing effect on blood vessels. However, the cyclooxygenase inhibitor indomethacin had no significant effect on the responses to GLP-1. Denudation or pretreatment with L-NAME significantly reduced GLP-1-induced relaxation to varying degrees, suggesting that these vasorelaxant effects are endothelium-dependent (Figure 3(a)). Furthermore, inhibitors were added to investigate which K^+^ channel is involved in the relaxation induced by GLP-1. GLP-1-induced relaxation was moderated by Glyb but was not affected by either 4-AP or Iber. Pretreatment with Glyb reduced GLP-1-induced relaxation by 43.39% (Figure 3(b)).

### 3.4. Effects of GLP-1 on K_ATP_ Currents in RCASMCs

**Figure 4:**
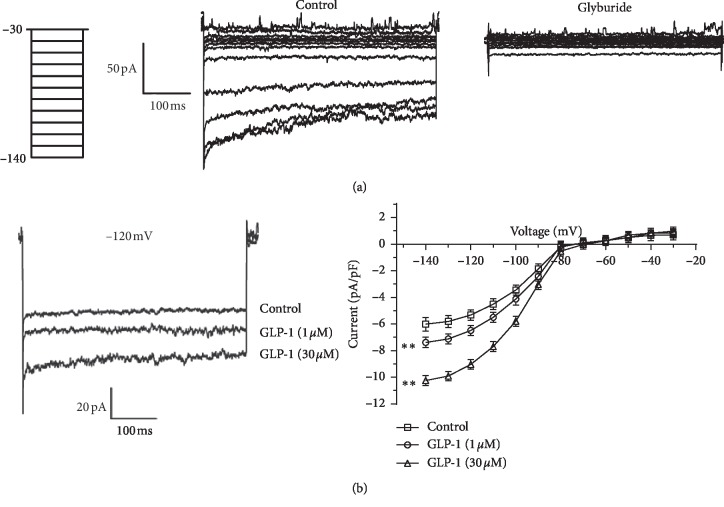
Effect of glucagon-like peptide-1 (GLP-1) on K_ATP_ currents in vascular smooth muscle cells freshly isolated from the rat coronary artery (RCASMCs). (a) Original recordings of the outward currents evoked by a series of depolarizing pulses (from −140 mV to −30 mV in 10 mV increments; duration 100 ms) in the absence or presence of the K_ATP_ channel blocker glyburide (1 *μ*M). (b) Left: recordings of the outward currents evoked by a test pulse (−120 mV) before and after treatment with GLP-1 (1 and 30 *μ*M). Right: I-V curves of K_ATP_ currents before and after application of GLP-1. Results are mean ± standard deviation indicated by the vertical bars; *n* = 6; ^*∗∗*^*P* < 0.01 vs. control.

K_ATP_ channel is one of the most significant potassium channels in adjusting vascular tone. Augmented activity of K_ATP_ channels may be involved in GLP-1-induced coronary relaxation. The K_V_ and K_Ca_ currents were minimized in the present patch clamp experiments to explore the K_ATP_ current separately. The K_ATP_ channel specific inhibitor Glyb remarkably blunted the outward K^+^ currents, suggesting that the outward K^+^ currents were supposed to occur primarily through K_ATP_ channels (Figure 4(a)). At a testing potential of −140 mV, the stable peak current was 158.4 ± 18.3 pA and current density was −6.04 ± 0.53 pA/pF; GLP-1 enhanced K_ATP_ currents in a concentration-dependent manner in RCASMCs (Figure 4(b), right). At a test potential of −120 mV, GLP-1 (1 and 30 *μ*M) increased the K_ATP_ currents by 17.9% and 41.2%, respectively (Figure 4(b), left).

## 4. Discussion

The effects of GLP-1 on coronary arteries were investigated from the perspectives of myogenicity and electrophysiology in this study. The main findings are as follows: (1) GLP-1 alleviated the RCA contractions induced by KCl and U46619; (2) the relaxation effect of RCA induced by GLP-1 was attenuated when intervening with Glyb, L-NAME, and denudation, respectively, but it was not affected by 4-AP, Iber, or Indo; (3) GLP-1 enhanced the K_ATP_ current in a concentration-dependent manner in RCASMCs.

GLP-1 is produced mainly in endocrine cells located in the intestine. GLP-1 controls glucose homeostasis by accelerating insulin secretion in a glucose-dependent manner, but it is not prone to a hypoglycemia risk compared with traditional hypoglycemic drugs because of its modulation of glucose-dependent insulin secretion. The active isoform of GLP-1 is a 30 amino acid peptide, GLP-1 (7–36), which binds to the classical GLP-1R and then activates a downstream signaling cascade [2]. As GLP-1R is expressed in many tissues and organs, the pleiotropic effects of GLP-1, apart from its hypoglycemic action, have been reported widely [2]. Increasing evidence has demonstrated that GLP-1 plays a crucial role in improving ventricular function, increasing cardiac output [21], reducing infarct size [22], and protecting against ischemic cardiac injury [23, 24]. Furthermore, experimental evidence on the *ex vivo* vascular actions reveals that GLP-1 (7–36) exerts a vasorelaxant action on different types of blood vessels, such as the rat thoracic aorta [5, 6], pulmonary artery [8], and femoral artery [9]. We found that GLP-1 also exerts a vasodilatory effect on rat coronary arteries. These experimental data may explain the ability of GLP-1 to promote coronary flow [1, 10] and cardiovascular benefits.

Additionally, different drugs were used to explore the possible mechanisms underlying the vasorelaxant effect of GLP-1 in RCAs. It is well known that vascular relaxation is associated with endothelial function as it produces endothelial-derived relaxing factors, particularly nitric oxide (NO) [25]. As a free radical gas, NO, generated by endothelial nitric oxide synthase (eNOS), catalyzes oxygen and L-arginine and plays a major role in ensuring normal vascular functioning [25]. Some studies suggest that GLP-1 analogues or DPP-4 inhibitors improve endothelial functioning by promoting activation of eNOS and enhancing NO production to prevent the progression of hypertension [26, 27] or atherosclerosis [28]. The vascular endothelium was involved in the relaxant effect of GLP-1 on the rat coronary artery. This result is consistent with earlier *ex vivo* studies about the effect of GLP-1 on the rat pulmonary artery [8, 29]. However, these findings are contradictory to other reports in which neither an eNOS inhibitor nor mechanical removal of the vascular endothelium affects the relaxant action induced by GLP-1 in the rat femoral artery [9] or aorta [5]. The reasons for the marked discrepancy remain unclear, but one possible interpretation is that this discrepancy may be relevant to different arterial rings and different experimental conditions.

K^+^ channels govern membrane conductance at rest and determine the resting membrane potential of RCASMCs. Therefore, multiple K^+^ channel openers usually serve as therapeutic vasodilators because of their indirect inhibition of the Ca^2+^ influx, which is produced by hyperpolarizing the resting membrane potential [30]. Different types of K^+^ channels are expressed in RCASMCs, but it seems that they regulate the contraction of coronary arteries to different degrees. For example, the nonselective potassium channel blocker tetraethylammonium or 4-AP causes strong contractions in coronary artery rings, whereas the K_Ca_ channel blockers charybdotoxin, Iber, and Glyb cause little or no contraction with or without the endothelium [19]. Nevertheless, other studies have shown that infusing Glyb into the coronary vascular bed lowers regional coronary flow and increases coronary perfusion pressure [30, 31]. The present results demonstrate that GLP-1-induced relaxation was inhibited by Glyb but not by other K^+^ channel blockers. These data suggest that the K_ATP_ channel is involved in the vasodilatation of GLP-1 in the RCA.

It has been reported previously that GLP-1 mediates the closure of K_ATP_ channels in pancreatic beta cells; this induces membrane depolarization and promotes Ca^2+^ influx, ultimately stimulating insulin secretion [32, 33]. The stimulation of the K_ATP_ signaling pathway in beta cells by GLP-1 in vascular cells, particularly the coronary artery, would be contradictory to its vasorelaxation effect. To further investigate the participation of K_ATP_ in vasorelaxation, the effect of GLP-1 on K_ATP_ currents in RCASMCs was studied with the whole-cell patch clamp technique. The results showed that GLP-1 increased the K_ATP_ current in a concentration-dependent manner in RCASMCs. This is the first study to demonstrate that enhancing K_ATP_ contributes to GLP-1-mediated vasodilation in RCAs. The different roles of GLP-1 in RCASMCs and beta cells may be due to the different expression of the K_ATP_ subunits in different cells.

**Figure 5:**
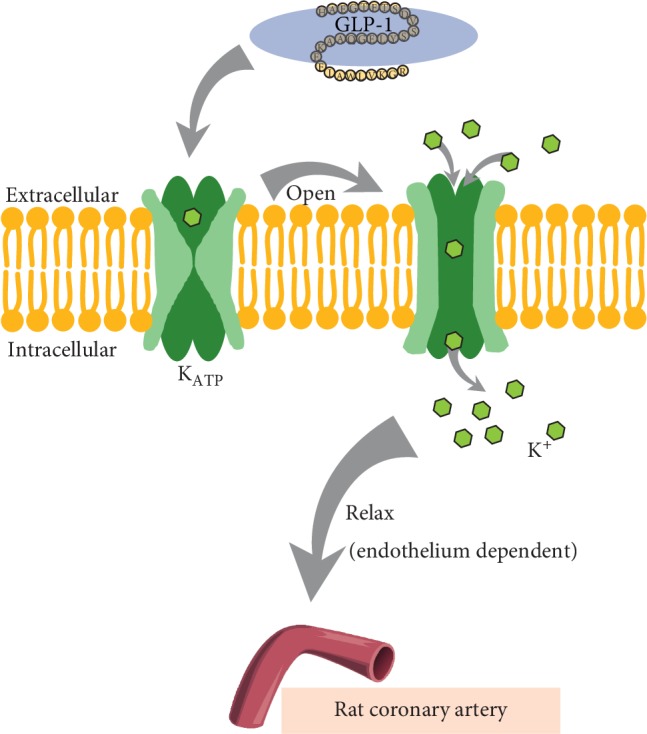
Proposed mechanism of the vasorelaxation effect of GLP-1 on the rat coronary artery. GLP-1 increases the K_ATP_ current in RCASMCs, which may be one of the causes of the vasodilation effect of GLP-1 on the rat coronary artery, and its vasodilation effect is endothelium-dependent.

Overall, the present study showed that GLP-1 is vasospasmolytic in RCAs. The vasorelaxant effects were endothelium-dependent and contributed to the enhancement of the K_ATP_ channels. Further studies are required to investigate the precise pathway mediating the opening of K_ATP_ by GLP-1 in RCASMCs (Figure 5). The direct vasorelaxant effect of GLP-1 suggests that it offers promise as an agent to provide an additional cardiovascular benefit beyond its hypoglycemic effect.

## 5. Limitations of Study

The present study has several limitations. First of all, although the results suggest that GLP-1 can enhance K_ATP_ currents, the potential mechanisms underlying GLP-1's opening effect should be investigated in the future. Secondly, whether the vasodilatory actions of GLP-1 are mediated by GLP-1R remains uncertain. At last, the dose of GLP-1 used in the present study is much higher compared with the physiological level in human. Therefore, it is meaningful to study the difference between rodents and humans. In addition, further studies are supposed to focus on the effect of GLP-1 on other types of blood vessels, such as the middle cerebral artery and mesenteric artery.

## Data Availability

The data used to support the findings of this study are available from the corresponding author upon request.
